# Idiopathic Scoliosis: Novel Challenges for Researchers and Clinicians

**DOI:** 10.3390/children10010103

**Published:** 2023-01-04

**Authors:** Fabio Zaina, Sabrina Donzelli, Stefano Negrini

**Affiliations:** 1 ISICO (Italian Scientific Spine Institute), 20141 Milan, Italy; 2 Department of Biomedical, Surgical and Dental Sciences, University “La Statale”, 20122 Milan, Italy; 3 IRCCS Istituto Ortopedico Galeazzi, 20157 Milan, Italy

Scoliosis is a three-dimensional deformity of the spine and trunk [1], and despite a well-documented genetic predisposition, the aetiology remains obscure, with experts considering it a multifactorial pathology [2]. Some studies have documented and described its progression being mainly due to biomechanical factors [3]. This led to the impossibility of any etiological treatment, making it mandatory to act on its consequences, trying to correct the deformity. Infancy and adolescence are the most challenging periods for potential scoliosis progression due to rapid growth [4]. Treatments aim to guide vertebral growth, revert the deformity and recover a more physiological vertebral and trunk morphology [1].

High-quality studies have expressed doubts about the possibility of bracing to change the natural history in mild scoliosis patients [5,6]. Some evidence has recently shown that there is a chance to also reduce the need for a surgical approach in surgical curves when patients are committed to a high-quality conservative treatment [7]. For this purpose, serial casting also seems to have a role [8], even if super rigid braces can guarantee similar results and better quality of life [9].

Evidence is also growing regarding exercises, with former Cochrane Review results [10] reporting that the currently very low-quality evidence for this treatment is expected to change. A recent systematic review reported that physiotherapeutic scoliosis-specific exercises (PSSEs) can reduce spinal deformities and improve quality of life as an isolated treatment or when administered in addition to bracing [11].

While we wait for a deeper understanding of the aetiology that would allow us a more specific treatment, based on current evidence, there are novel challenges that clinicians and researchers must face. After focusing on effectiveness for decades, research concerning bracing should now focus on protocols and biomechanical effectiveness. The high dosages (hours per day of brace wearing) are a documented key factor for success [12]. However, details about the weaning phase, the role of exercise [13], materials and design are still scant, with few papers directly comparing different approaches [14]. A novel classification of bracing [15] has been developed and could act as a starting point for this novel challenge, together with shared guidelines on brace management [16] and building [17]. Moreover, the results reported in the current literature are very different due to factors such as efficacy and the quality of the braces, as well as the compliance of patients [15]. Another obstacle that makes a comparison challenging is that when effectiveness is very low, for whatever reason (compliance issues, low number of hours prescribed, poor quality braces, etc.), all braces seem to have the same effects [18,19]. Defining the minimum required efficacy of braces for research studies could be another crucial advancement.

Evidence for PSSEs is quite promising [11], with novel methods based on small changes to existing ones appearing and, therefore, making it more complicated for clinicians to interpret and apply data from the literature. The current consensus about the fundamental role of active self-correction in PSSEs [20] should probably be the standard base for a comprehensive approach, including all the schools supported by published data [21]. Future improvements should be built on such a comprehensive approach and shared within the research and clinical community, and not be the starting point for the purpose of splitting it into different methods.

Poor-quality research remains crucial in limiting the possibility of increasing our knowledge and implementing the best available treatments. Several years ago, we described some of the most frequent issues related to this, including selection bias, inappropriate outcome measures and follow-ups, the misinterpretation of findings and missing evaluations of skeletal maturity [22]. These problems seem far from having their solutions realized, and misleading information could also affect secondary research (reviews and meta-analyses), where systematic reviews state they include scoliosis patients when in fact they do not [23].

In conclusion, in recent decades, knowledge on idiopathic scoliosis and its treatment has grown thanks to broader interest in and better quality of research. The creation of the Scientific Society on Scoliosis Orthopaedic and Rehabilitation Treatment (SOSORT) strongly supported the increased number of published papers. Nevertheless, some areas are still under investigation, and poor-quality articles are still being published. We need to focus on these areas and provide the best possible evidence for the sake of our patients. The present Special Issue is an example of interest in bridging this gap between current knowledge and the needs of clinicians.
